# Supplementary material for: Glial reactivity and cognitive decline follow chronic heterochromatin loss in neurons
Source: Nat Commun. 2025 Aug 8;16:7325. doi: 10.1038/s41467-025-61319-7 (PMC12334701; doi:10.1038/s41467-025-61319-7)
Supplement: Supplementary file 2 — Description of Additional Supplementary Files [file 41467_2025_61319_MOESM2_ESM.docx]

**Description of Additional Supplementary Files**

**Supplementary Data 1:** Differential gene expression (Total RNA, EdgeR), Chimeric transcripts, primers, antibodies, sequencing depth and alignment statistics.

**Supplementary Data 2:** Locus specific analysis of Transposable elements (SalmonTE*) table including counts, statistics and annotations.

**Supplementary Data 3:** Report from GSEA analysis of Aged HP1DKO hippocampi (bulk RNAseq, TETranscripts analysis)
