# Supplementary material for: Glial reactivity and cognitive decline follow chronic heterochromatin loss in neurons
Source: Nat Commun. 2025 Aug 8;16:7325. doi: 10.1038/s41467-025-61319-7 (PMC12334701; doi:10.1038/s41467-025-61319-7)
Supplement: Supplementary file 6 — Reporting Summary [file 41467_2025_61319_MOESM6_ESM.pdf]

Corresponding author(s): Andrew Newman
Last updated by author(s): Jun 2, 2025

Reporting Summary

Nature Portfolio wishes to improve the reproducibility of the work that we publish. This form provides structure for consistency and transparency in reporting. For further information on Nature Portfolio policies, see our Editorial Policies and the Editorial Policy Checklist.

Statistics

For all statistical analyses, confirm that the following items are present in the figure legend, table legend, main text, or Methods section.

n/a	Confirmed	
☐	☒	The exact sample size (*n*) for each experimental group/condition, given as a discrete number and unit of measurement
☐	☒	A statement on whether measurements were taken from distinct samples or whether the same sample was measured repeatedly
☐	☒	The statistical test(s) used AND whether they are one- or two-sided<br>*Only common tests should be described solely by name; describe more complex techniques in the Methods section.*
☐	☒	A description of all covariates tested
☐	☒	A description of any assumptions or corrections, such as tests of normality and adjustment for multiple comparisons
☐	☒	A full description of the statistical parameters including central tendency (e.g. means) or other basic estimates (e.g. regression coefficient) AND variation (e.g. standard deviation) or associated estimates of uncertainty (e.g. confidence intervals)
☐	☒	For null hypothesis testing, the test statistic (e.g. *F*, *t*, *r*) with confidence intervals, effect sizes, degrees of freedom and *P* value noted<br>*Give P values as exact values whenever suitable.*
☒	☐	For Bayesian analysis, information on the choice of priors and Markov chain Monte Carlo settings
☒	☐	For hierarchical and complex designs, identification of the appropriate level for tests and full reporting of outcomes
☒	☐	Estimates of effect sizes (e.g. Cohen's *d*, Pearson's *r*), indicating how they were calculated

Our web collection on statistics for biologists contains articles on many of the points above.

Software and code

Policy information about availability of computer code

Data collection	No software was used for Data collection
Data analysis	Data was analyzed using open source software and plotted using Rstudio.<br>Images and figures were formatted using Adobe Photoshop and Adobe Illustrator.

For manuscripts utilizing custom algorithms or software that are central to the research but not yet described in published literature, software must be made available to editors and reviewers. We strongly encourage code deposition in a community repository (e.g. GitHub). See the Nature Portfolio guidelines for submitting code & software for further information.

Data

Policy information about availability of data

All manuscripts must include a data availability statement. This statement should provide the following information, where applicable:

- Accession codes, unique identifiers, or web links for publicly available datasets
- A description of any restrictions on data availability
- For clinical datasets or third party data, please ensure that the statement adheres to our policy

Data availability: The sequencing data from this study has been deposited at GEO database under accession # GSE153331. ZFP57 Binding sites were obtained from GSE77444. Raw Data from figures and statistics from this work can be found in the Source Data file.
Code availability: Code used in analysis can be accessed at https://github.com/qoldt/HP1-Deficiency-Neurodegeneration

Research involving human participants, their data, or biological material

Policy information about studies with human participants or human data. See also policy information about sex, gender (identity/presentation), and sexual orientation and race, ethnicity and racism.

Reporting on sex and gender	*Use the terms sex (biological attribute) and gender (shaped by social and cultural circumstances) carefully in order to avoid confusing both terms. Indicate if findings apply to only one sex or gender; describe whether sex and gender were considered in study design; whether sex and/or gender was determined based on self-reporting or assigned and methods used. Provide in the source data disaggregated sex and gender data, where this information has been collected, and if consent has been obtained for sharing of individual-level data; provide overall numbers in this Reporting Summary. Please state if this information has not been collected. Report sex- and gender-based analyses where performed, justify reasons for lack of sex- and gender-based analysis.*
Reporting on race, ethnicity, or other socially relevant groupings	*Please specify the socially constructed or socially relevant categorization variable(s) used in your manuscript and explain why they were used. Please note that such variables should not be used as proxies for other socially constructed/relevant variables (for example, race or ethnicity should not be used as a proxy for socioeconomic status). Provide clear definitions of the relevant terms used, how they were provided (by the participants/respondents, the researchers, or third parties), and the method(s) used to classify people into the different categories (e.g. self-report, census or administrative data, social media data, etc.) Please provide details about how you controlled for confounding variables in your analyses.*
Population characteristics	*Describe the covariate-relevant population characteristics of the human research participants (e.g. age, genotypic information, past and current diagnosis and treatment categories). If you filled out the behavioural & social sciences study design questions and have nothing to add here, write "See above."*
Recruitment	*Describe how participants were recruited. Outline any potential self-selection bias or other biases that may be present and how these are likely to impact results.*
Ethics oversight	*Identify the organization(s) that approved the study protocol.*

Note that full information on the approval of the study protocol must also be provided in the manuscript.

Field-specific reporting

Please select the one below that is the best fit for your research. If you are not sure, read the appropriate sections before making your selection.

☒ Life sciences ☐ Behavioural & social sciences ☐ Ecological, evolutionary & environmental sciences

For a reference copy of the document with all sections, see nature.com/documents/nr-reporting-summary-flat.pdf

Life sciences study design

All studies must disclose on these points even when the disclosure is negative.

Sample size	Assuming a low coefficient of variation between tissue samples, for RNAseq experiments, 3-4 replicates per condition at ~25 million read depth would achieve sufficient power to reject the null For behavioural experiments, sample size was calculated to be higher to account for higher variation between individuals.
Data exclusions	The sequencing run from aged BKO replicate 3 largely failed and has been omitted.
Replication	Where possilbe, all experiments were successfully replicated a second time at a later date.
Randomization	Behavioural experiments maintained animals of all genotypes in mixed cages. Within batch NGS experiments loaded all genotypes across lanes
Blinding	Behavioural experiments were performed blind, using only the animal number.

Reporting for specific materials, systems and methods

We require information from authors about some types of materials, experimental systems and methods used in many studies. Here, indicate whether each material, system or method listed is relevant to your study. If you are not sure if a list item applies to your research, read the appropriate section before selecting a response.

Materials & experimental systems

n/a	Involved in the study
[ ]	[x] Antibodies
[ ]	[x] Eukaryotic cell lines
[x]	[ ] Palaeontology and archaeology
[ ]	[x] Animals and other organisms
[x]	[ ] Clinical data
[x]	[ ] Dual use research of concern
[x]	[ ] Plants

Methods

n/a	Involved in the study
[ ]	[x] ChIP-seq
[x]	[ ] Flow cytometry
[ ]	[x] MRI-based neuroimaging

Antibodies

Antibodies used

rabbit anti-HP1α (N term) LS-C286285 LSBio
mouse anti-HP1α (N term) MAB3446 Millipore
mouse anti-HP1β MAB3448 Millipore
rat anti-HP1β Self-Made by Singh laboratory
mouse anti-HP1γ MAB3450 Millipore
rabbit anti-H3K9me3 Self-Made by Singh laboratory
rabbit anti-H4K20me3 Self-Made by Singh laboratory
rabbit anti-ki67 Self-Made by Singh laboratory
rabbit anti-Satb2 Self-Made by Tarabykin laboratory
rat anti-Ctip2 ab18465 Abcam
goat anti-GFP 600-101-215 Rockland
chicken anti-GFP ab13970 Abcam
goat anti-Prox1 AF2727 R\&D Systems
goat anti-tdTomato AB8181-200 SICGEN Antibodies
mouse anti-Myc (9B11) 2276 S Cell Signalling
rabbit anti-Iba1 019-19741 wako
mouse anti-GFAP MAB360 Millipore
rat anti-CD68 MCA1957T Bio-Rad
mouse anti-KAP1 (20C1) GTX80695 GeneTex
mouse anti-Histone H3K9me3, clone: Clone: MABI 0319 61014 Active Motif
mouse Anti-Histone H4K20me3 (trimethyl Lys20) (GT9009) GTX60364 GeneTex
goat anti-RANTES AF478 bio-techne
goat anti-C3 55730 MP bio
rabbit anti-IRF7 72073 Cell Signalling
rabbit anti-RIG-1 3743 Cell signalling
rabbit anti-ISG15 2743S Cell Signalling

Validation

Describe the validation of each primary antibody for the species and application, noting any validation statements on the manufacturer's website, relevant citations, antibody profiles in online databases, or data provided in the manuscript.

Eukaryotic cell lines

Policy information about cell lines and Sex and Gender in Research

Cell line source(s): The HP1cTKO cell line is derived from Mouse embryonic stem cells

Authentication: Engineering of HP1cTKO was confirmed by PCR. Tamoxifen-mediated deletion of Cbx1, Cbx3 and Cbx5 was confirmed by negative antibody staining.

Mycoplasma contamination: Cells are regularly checked for mycoplasma contamination

Commonly misidentified lines (See ICLAC register): *Name any commonly misidentified cell lines used in the study and provide a rationale for their use.*

Animals and other research organisms

Policy information about studies involving animals; ARRIVE guidelines recommended for reporting animal research, and Sex and Gender in Research

Laboratory animals: Young adult male HP1FEC mice (3-4 months) and middle aged adult male HP1FEC mice (12-13 months) were used in this study.

Wild animals: *Provide details on animals observed in or captured in the field; report species and age where possible. Describe how animals were caught and transported and what happened to captive animals after the study (if killed, explain why and describe method; if released,*

	say where and when) OR state that the study did not involve wild animals.
Reporting on sex	The findings in this study are generalizable across sex. Given 4 genotypes across 2 ages we could not feasibly test both sexes in all our experiments. For consistency sequencing experiments and behavioural experiments were performed in males only. In situ hybridization could confirm de-repression of ERV transcripts in HP1DKO brains regardless of sex.
Field-collected samples	*For laboratory work with field-collected samples, describe all relevant parameters such as housing, maintenance, temperature, photoperiod and end-of-experiment protocol OR state that the study did not involve samples collected from the field.*
Ethics oversight	Housing and behavioural experiments on HP1FEC mice was carried out in accordance with LaGeSo guidelines and German law.

Note that full information on the approval of the study protocol must also be provided in the manuscript.

Plants

Seed stocks	*Report on the source of all seed stocks or other plant material used. If applicable, state the seed stock centre and catalogue number. If plant specimens were collected from the field, describe the collection location, date and sampling procedures.*
Novel plant genotypes	*Describe the methods by which all novel plant genotypes were produced. This includes those generated by transgenic approaches, gene editing, chemical/radiation-based mutagenesis and hybridization. For transgenic lines, describe the transformation method, the number of independent lines analyzed and the generation upon which experiments were performed. For gene-edited lines, describe the editor used, the endogenous sequence targeted for editing, the targeting guide RNA sequence (if applicable) and how the editor was applied.*
Authentication	*Describe any authentication procedures for each seed stock used or novel genotype generated. Describe any experiments used to assess the effect of a mutation and, where applicable, how potential secondary effects (e.g. second site T-DNA insertions, mosiacism, off-target gene editing) were examined.*

ChIP-seq

Data deposition

- [x] Confirm that both raw and final processed data have been deposited in a public database such as GEO.
- [x] Confirm that you have deposited or provided access to graph files (e.g. BED files) for the called peaks.

Data access links<br>*May remain private before publication.*	https://www.ncbi.nlm.nih.gov/geo/query/acc.cgi?acc=GSE153331
Files in database submission	*Provide a list of all files available in the database submission.*
Genome browser session<br>(e.g. UCSC)	*Provide a link to an anonymized genome browser session for "Initial submission" and "Revised version" documents only, to enable peer review. Write "no longer applicable" for "Final submission" documents.*

Methodology

Replicates	Each ChIPseq was performed with a replicate in a line derived from a separate ES clone
Sequencing depth	Exact Sequencing depth and alignment efficiency is reported in Data S1
Antibodies	anti-KAP1, mouse-mono(20C1), GTX80695<br>Histone H3K9me3 antibody (mAb), Clone: MABI 0319<br>Anti-Histone H4K20me3 (trimethyl Lys20), Mouse(GT9009), GTX60364
Peak calling parameters	Peaks were not called, ChIP signal over repeats was measured using published annotations (UCSC table browser, repeatmasker)
Data quality	*Describe the methods used to ensure data quality in full detail, including how many peaks are at FDR 5% and above 5-fold enrichment.*
Software	ChIPseq reads were trimmed using trimmomatic SE with LEADING:5, TRAILING:3 SLIDINGWINDOW:4:15 MINLEN:30.<br>Trimmed reads were aligned using bowtie2 using default parameters. Bigwig files were created using deeptools' bamCoverage with --normalizeUsing RPGC --effectiveGenomeSize 2652783500 --extendReads 125

Magnetic resonance imaging

Experimental design

Design type	Volume of whole brain was measured immediately post-mortem
Design specifications	Animals from each genotype were measured across 3 cohorts, with each cohort taking 1-2 days to scan the animals of that cohort.

Behavioral performance measures

State number and/or type of variables recorded (e.g. correct button press, response time) and what statistics were used to establish that the subjects were performing the task as expected (e.g. mean, range, and/or standard deviation across subjects).

Acquisition

Imaging type(s): Structural

Field strength: 3T

Sequence & imaging parameters: T2-weighted 2D turbo spin-echo sequence , TR/TE = 5505 ms/36 ms, rare factor 8, 6 averages,
46 axial slices with a slice thickness of 0.350 mm, field of view of 2.56 x 2.56 cm, matrix size 256 x 256; scan time 13m12s

Area of acquisition: Whole mouse brain

Diffusion MRI ☐ Used ☒ Not used

Preprocessing

Preprocessing software

Provide detail on software version and revision number and on specific parameters (model/functions, brain extraction, segmentation, smoothing kernel size, etc.).

Normalization

If data were normalized/standardized, describe the approach(es): specify linear or non-linear and define image types used for transformation OR indicate that data were not normalized and explain rationale for lack of normalization.

Normalization template

Describe the template used for normalization/transformation, specifying subject space or group standardized space (e.g. original Talairach, MNI305, ICBM152) OR indicate that the data were not normalized.

Noise and artifact removal

Describe your procedure(s) for artifact and structured noise removal, specifying motion parameters, tissue signals and physiological signals (heart rate, respiration).

Volume censoring

Define your software and/or method and criteria for volume censoring, and state the extent of such censoring.

Statistical modeling & inference

Model type and settings

Specify type (mass univariate, multivariate, RSA, predictive, etc.) and describe essential details of the model at the first and second levels (e.g. fixed, random or mixed effects; drift or auto-correlation).

Effect(s) tested

Define precise effect in terms of the task or stimulus conditions instead of psychological concepts and indicate whether ANOVA or factorial designs were used.

Specify type of analysis: ☐ Whole brain ☐ ROI-based ☐ Both

Statistic type for inference

(See Eklund et al. 2016)

Specify voxel-wise or cluster-wise and report all relevant parameters for cluster-wise methods.

Correction

Describe the type of correction and how it is obtained for multiple comparisons (e.g. FWE, FDR, permutation or Monte Carlo).

Models & analysis

n/a	Involved in the study
☒	☐ Functional and/or effective connectivity
☒	☐ Graph analysis
☒	☐ Multivariate modeling or predictive analysis
