# Supplementary material for: Glial reactivity and cognitive decline follow chronic heterochromatin loss in neurons
Source: Nat Commun. 2025 Aug 8;16:7325. doi: 10.1038/s41467-025-61319-7 (PMC12334701; doi:10.1038/s41467-025-61319-7)

Supplementary Information

Glial Reactivity and Cognitive Decline Follow Chronic Heterochromatin Loss in Neurons

A.G. Newman*, J. Sharif, P. Bessa, S. Zaqout, J. P. Brown, D. Richter, R. Dannenberg, M., Nakayama, S. Mueller, T. Schaub, S. Manickaraj, P. Böhm-Sturm, O. Ohara, H. Koseki, P.B. Singh[#,]*, V. Tarabykin[#,]*

[#]These Authors Jointly supervised this work
*For Correspondence: andrew.newman@pm.me (A.G.N); prim.singh@nu.edu.kz (P.B.S.); tarabykinvictor@gmail.com (V.T).

Contents

Graphical Summary
Supplementary Figure 1
Supplementary Figure 2
Supplementary Figure 3
Supplementary Figure 4
Supplementary Figure 5
Supplementary Figure 6
Supplementary Figure 7
Supplementary Figure 8
Supplementary Figure 9
Supplementary Figure 10
Supplementary Figure 11
Supplementary Figure 12
Full Membranes from Western Blots (figure 2)
Full Membranes from Western Blots (figure 6)

Graphical Summary

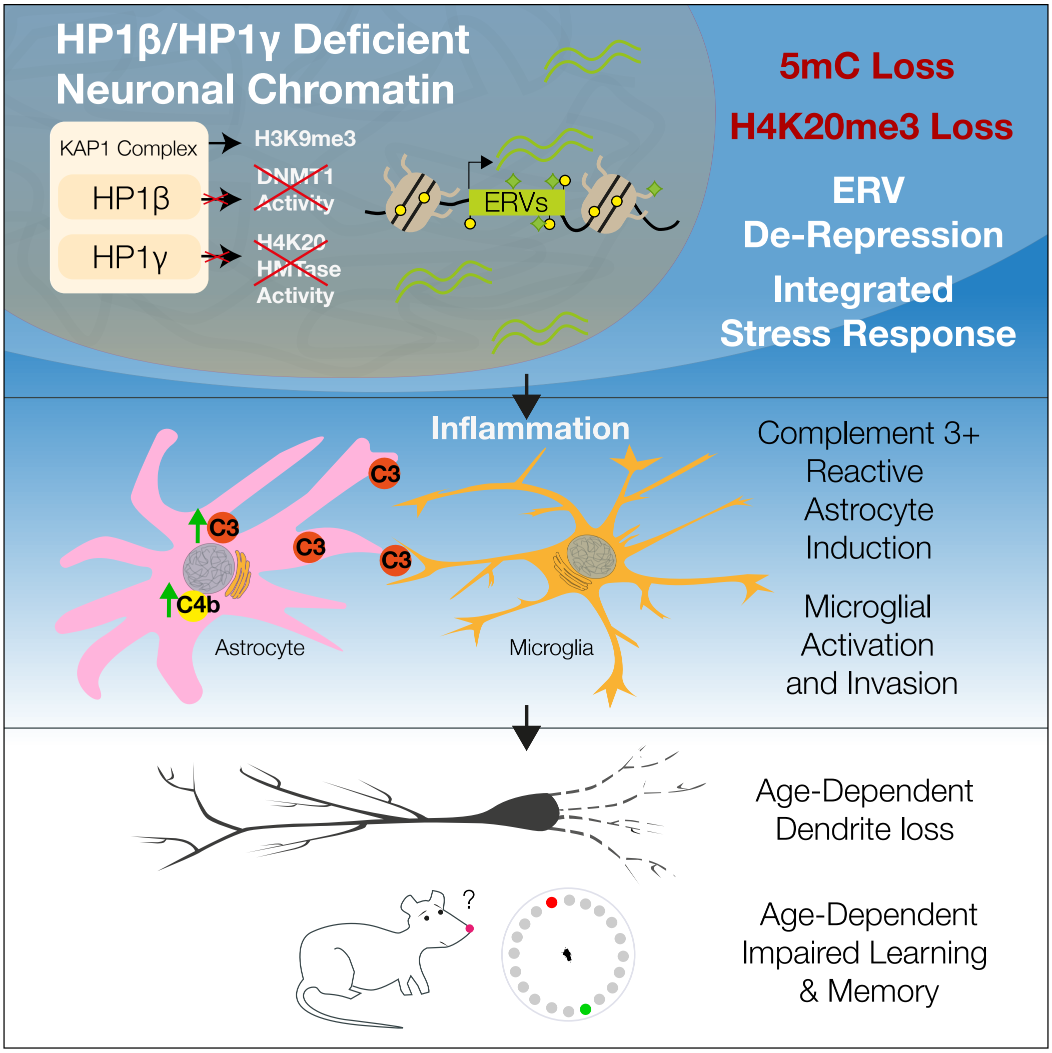

Figure S1

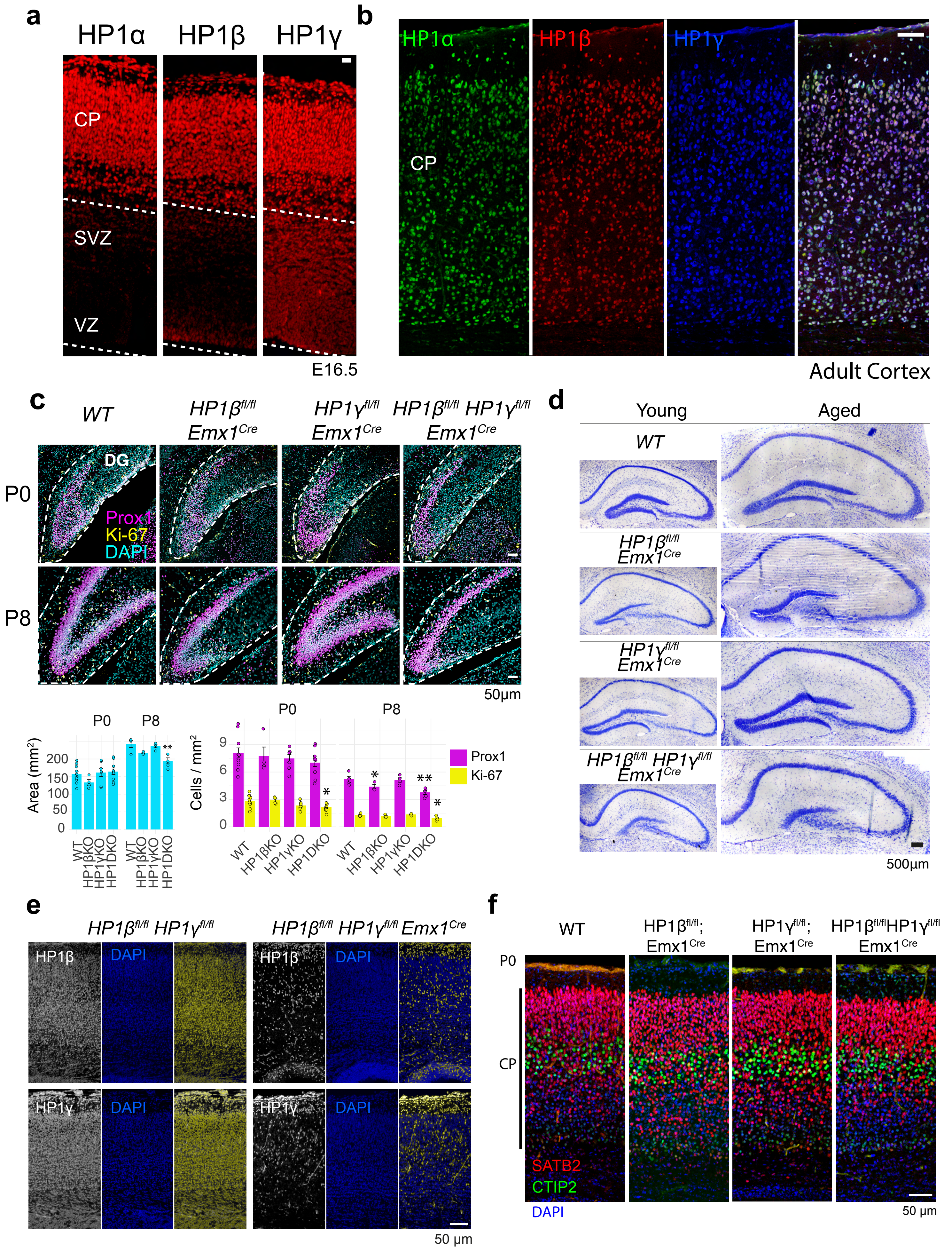
a
HP1α
HP1β
HP1γ
CP
SVZ
VZ
E16.5
b
HP1α
HP1β
HP1γ
CP
Adult Cortex
c
WT
HP1β fl/fl Emx1Cre
HP1γ fl/fl Emx1Cre
HP1β fl/fl HP1γ fl/fl Emx1Cre
P0
P8
DG
Prox1
Ki-67
DAPI
50μm
P0
P8
Area (mm²)
WT
HP1βKO
HP1γKO
HP1DKO
Cells / mm²
Prox1
Ki-67
d
Young
Aged
WT
HP1β fl/fl Emx1Cre
HP1γ fl/fl Emx1Cre
HP1β fl/fl HP1γ fl/fl Emx1Cre
500μm
e
HP1β fl/fl HP1γ fl/fl
HP1β fl/fl HP1γ fl/fl Emx1Cre
HP1β
HP1γ
DAPI
50 μm
f
WT
HP1β fl/fl; Emx1Cre
HP1γ fl/fl; Emx1Cre
HP1β fl/fl HP1γ fl/fl Emx1Cre
P0
CP
SATB2
CTIP2
DAPI
50 μm

Figure S1 (Previous Page)

Expression of HP1 proteins and deletion from the cerebral cortex. **a** HP1α and HP1β expression is strongest in post-mitotic neurons of the cortical plate (CP) at embryonic day 16.5. Both are also expressed in progenitor cells in the Subventricular and ventricular zones (SVZ and VZ, respectively). HP1γ expression is much more uniformly expressed across progenitor and post-mitotic cell types. (scale bar = 50 μm) **b** HP1 proteins remain robustly expressed in the cortical plate of adult mice (scale bar = 100μm). **c** Dentate gyri of HP1βKO and HP1β/γKO animals show malformation of the infrapyramidal blade due to mitotic exhaustion between postnatal day 0 and postnatal day 8. P0 WT n = 10, P0 HP1βKO n = 4, P0 HP1γKO n = 7, P0 HP1DKO n= 11, P8 WT n = 4, P8 HP1βKO n = 3, P8 HP1γKO n = 4, P8 HP1DKO n= 5. One way ANOVA with Dunnett's Multiple Comparison test where ** denotes $p < 0.05$, and * denotes $p < 0.01$. **d** The morphology of young (3 month) and aged (13 month) hippocampi. **e** Emx1-Cre mediated deletion of floxed HP1 proteins results in targeted deletion of HP1β and HP1γ in the pyramidal lineage seen here at postnatal day 0 (P0). **f** Single or double deletion of HP1β or HP1γ does not adversely affect neuronal cell fate or laminal position in the cerebral cortex at P0. All values and exact p values can be seen in the Source Data file.

Figure S2

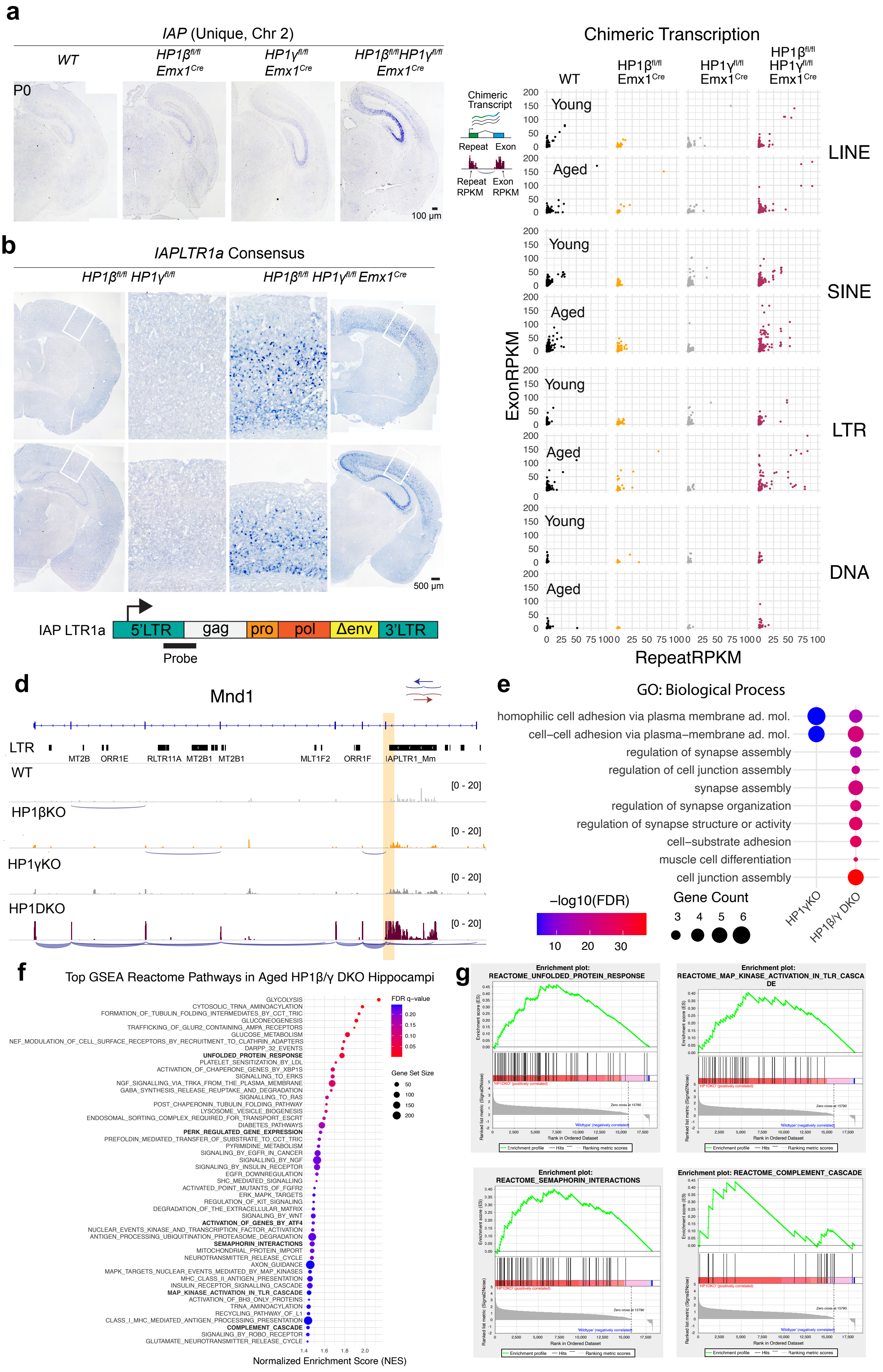
a
IAP (Unique, Chr 2)
WT
HP1β fl/fl Emx1 Cre
HP1γ fl/fl Emx1 Cre
HP1β fl/fl HP1γ fl/fl Emx1 Cre
P0
100 µm
b
IAPLTR1a Consensus
HP1β fl/fl HP1γ fl/fl
HP1β fl/fl HP1γ fl/fl Emx1 Cre
500 µm
IAP LTR1a
5'LTR
gag
pro
pol
Δenv
3'LTR
Probe
Chimeric Transcription
Chimeric Transcript
Repeat
Exon
Repeat RPKM
Exon RPKM
WT
HP1β fl/fl Emx1 Cre
HP1γ fl/fl Emx1 Cre
HP1β fl/fl HP1γ fl/fl Emx1 Cre
Young
Aged
LINE
SINE
LTR
DNA
ExonRPKM
RepeatRPKM
d
Mnd1
LTR
MT2B
ORR1E
RLTR11A
MT2B1
MT2B1
MLT1F2
ORR1F
IAPLTR1_Mm
WT
HP1βKO
HP1γKO
HP1DKO
[0 - 20]
e
GO: Biological Process
homophilic cell adhesion via plasma membrane ad. mol.
cell−cell adhesion via plasma−membrane ad. mol.
regulation of synapse assembly
regulation of cell junction assembly
synapse assembly
regulation of synapse organization
regulation of synapse structure or activity
cell−substrate adhesion
muscle cell differentiation
cell junction assembly
−log10(FDR)
10
20
30
Gene Count
3
4
5
6
HP1γKO
HP1β/γ DKO
f
Top GSEA Reactome Pathways in Aged HP1β/γ DKO Hippocampi
GLYCOLYSIS
CYTOSOLIC_TRNA_AMINOACYLATION
FORMATION_OF_TUBULIN_FOLDING_INTERMEDIATES_BY_CCT_TRIC
GLUCONEOGENESIS
TRAFFICKING_OF_GLUR2_CONTAINING_AMPA_RECEPTORS
GLUCOSE_METABOLISM
NEF_MODULATION_OF_CELL_SURFACE_RECEPTORS_BY_RECRUITMENT_TO_CLATHRIN_ADAPTERS
DARPP_32_EVENTS
UNFOLDED_PROTEIN_RESPONSE
PLATELET_SENSITIZATION_BY_LDL
ACTIVATION_OF_CHAPERONE_GENES_BY_XBP1S
SIGNALLING_TO_ERKS
NGF_SIGNALLING_VIA_TRKA_FROM_THE_PLASMA_MEMBRANE
GABA_SYNTHESIS_RELEASE_REUPTAKE_AND_DEGRADATION
SIGNALLING_TO_RAS
POST_CHAPERONIN_TUBULIN_FOLDING_PATHWAY
LYSOSOME_VESICLE_BIOGENESIS
ENDOSOMAL_SORTING_COMPLEX_REQUIRED_FOR_TRANSPORT_ESCRT
DIABETES_PATHWAYS
PERK_REGULATED_GENE_EXPRESSION
PREFOLDIN_MEDIATED_TRANSFER_OF_SUBSTRATE_TO_CCT_TRIC
PYRIMIDINE_METABOLISM
SIGNALING_BY_EGFR_IN_CANCER
SIGNALLING_BY_NGF
SIGNALING_BY_INSULIN_RECEPTOR
EGFR_DOWNREGULATION
SHC_MEDIATED_SIGNALLING
ACTIVATED_POINT_MUTANTS_OF_FGFR2
ERK_MAPK_TARGETS
REGULATION_OF_KIT_SIGNALING
DEGRADATION_OF_THE_EXTRACELLULAR_MATRIX
SIGNALING_BY_WNT
ACTIVATION_OF_GENES_BY_ATF4
NUCLEAR_EVENTS_KINASE_AND_TRANSCRIPTION_FACTOR_ACTIVATION
ANTIGEN_PROCESSING_UBIQUITINATION_PROTEASOME_DEGRADATION
SEMAPHORIN_INTERACTIONS
MITOCHONDRIAL_PROTEIN_IMPORT
NEUROTRANSMITTER_RELEASE_CYCLE
AXON_GUIDANCE
MAPK_TARGETS_NUCLEAR_EVENTS_MEDIATED_BY_MAP_KINASES
MHC_CLASS_II_ANTIGEN_PRESENTATION
INSULIN_RECEPTOR_SIGNALLING_CASCADE
MAP_KINASE_ACTIVATION_IN_TLR_CASCADE
ACTIVATION_OF_BH3_ONLY_PROTEINS
TRNA_AMINOACYLATION
RECYCLING_PATHWAY_OF_L1
CLASS_I_MHC_MEDIATED_ANTIGEN_PROCESSING_PRESENTATION
COMPLEMENT_CASCADE
SIGNALING_BY_ROBO_RECEPTOR
GLUTAMATE_NEUROTRANSMITTER_RELEASE_CYCLE
FDR q-value
Gene Set Size
Normalized Enrichment Score (NES)
g
Enrichment plot: REACTOME_UNFOLDED_PROTEIN_RESPONSE
Enrichment plot: REACTOME_MAP_KINASE_ACTIVATION_IN_TLR_CASCADE
Enrichment plot: REACTOME_SEMAPHORIN_INTERACTIONS
Enrichment plot: REACTOME_COMPLEMENT_CASCADE
Enrichment score (ES)
Ranked list metric (Signal2Noise)
Rank in Ordered Dataset
Enrichment profile
Hits
Ranking metric scores

Figure S2 (Previous Page)

Sustained de-repression of ERVs and induction of chimeric transcripts in HP1β/γDKO mutants. **a** In situ hybridization on P0 brains using an RNA probe specific for a single IAP element on chromosome 2. **b** In situ hybridization on adult brains using an RNA probe that recognizes an IAPLTR1a consensus sequence found on at least 196 IAPs. **c** Co-transcription plots of chimeric transcripts where each point represents a chimeric transcript. **d** An example chimeric transcript; transcriptional activation of an IAP element in the first intron of *Mnd1* results in transcriptional activation of the entire gene. Shown here are RNAseq tracks from aged genotypes. **e** Overrepresentation analysis of gene Ontology of significantly changed genes (edgeR FDR <0.05) in HP1βKO, HP1γKO, and HP1β/γKO RNAseq. No significant gene ontology could be observed for HP1βKO. The differential expression of protocadherins in HP1γKO and HP1β/γKO brains greatly biases gene ontology towards cell adhesion and synapse gene sets. **f** Top reactome pathways from Gene Set Enrichment Analysis (GSEA) from entire HP1! /γDKO transcriptome (Top 50 ordered by NES). **g** Leading edge plots for select Reactome pathways significantly changed in HP1DKO brains: Unfolded Protein Response, MAP kinase activation in TLR cascade, Semaphorin Interactions, and Complement Cascade.

Figure S3

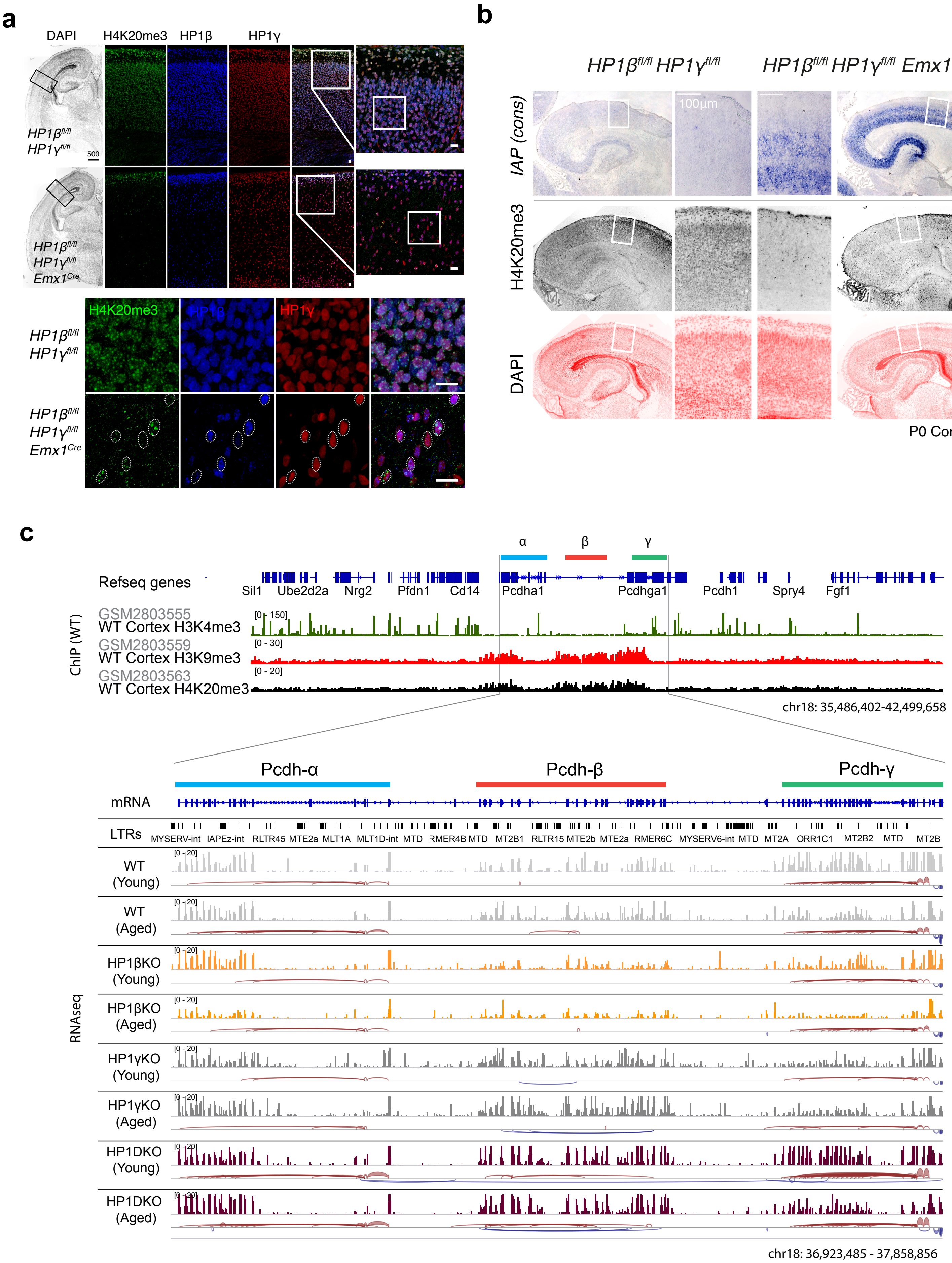
a
DAPI
H4K20me3
HP1β
HP1γ
HP1β fl/fl HP1γ fl/fl
HP1β fl/fl HP1γ fl/fl Emx1 Cre
500
b
HP1β fl/fl HP1γ fl/fl
HP1β fl/fl HP1γ fl/fl Emx1 Cre
IAP (cons)
H4K20me3
DAPI
100μm
P0 Cortex
c
α
β
γ
Refseq genes
Sil1
Ube2d2a
Nrg2
Pfdn1
Cd14
Pcdha1
Pcdhga1
Pcdh1
Spry4
Fgf1
ChIP (WT)
GSM2803555
WT Cortex H3K4me3
[0 - 150]
GSM2803559
WT Cortex H3K9me3
[0 - 30]
GSM2803563
WT Cortex H4K20me3
[0 - 20]
chr18: 35,486,402-42,499,658
Pcdh-α
Pcdh-β
Pcdh-γ
mRNA
LTRs
MYSERV-int IAPEz-int RLTR45 MTE2a MLT1A MLT1D-int MTD RMER4B MTD MT2B1 RLTR15 MTE2b MTE2a RMER6C MYSERV6-int MTD MT2A ORR1C1 MT2B2 MTD MT2B
RNAseq
WT (Young)
WT (Aged)
HP1βKO (Young)
HP1βKO (Aged)
HP1γKO (Young)
HP1γKO (Aged)
HP1DKO (Young)
HP1DKO (Aged)
[0 - 20]
chr18: 36,923,485 - 37,858,856

Figure S3 (Previous Page)

H4K20me3 loss is correlated with IAP de-repression and Protocadherin expression. H4K20me3, normally abundant in post-mitotic cortical neurons is completely missing in HP1γ deficient Emx1-lineage pyramidal neurons. In cortices missing HP1γ, H4K20me3 can still be seen in adjacent wild type interneurons (see magnifications). Unless stated, all scalebars 50µm. **b** *In situ hybridization* of IAP consensus sequence alongside adjacent sections stained for H4K20me3 and Dapi. **c** HP1γ is required for transcriptional regulation of the protocadherin cluster. (Top): Reference gene annotation of the mouse protocadherin α β and γ clusters on chromosome 18 along with published ChIPseq data of H3K4me3, H3K9me3 and H4K20me3 performed in cortical neurons [40]. (Bottom): mRNA and LTR reference annotation along with read coverage tracks from hippocampal RNAseq performed in this study.

Figure S4

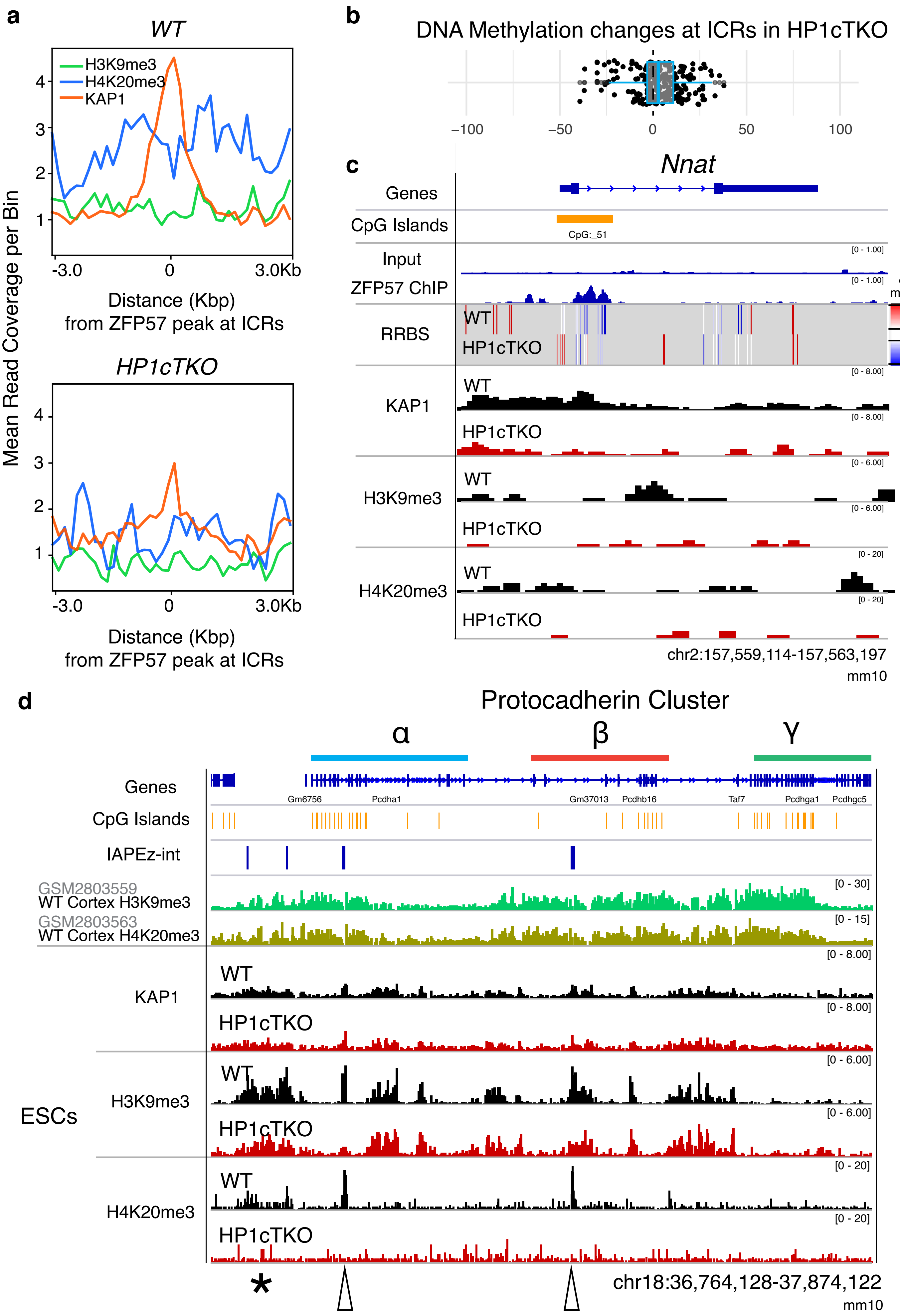
a
WT
H3K9me3
H4K20me3
KAP1
Mean Read Coverage per Bin
Distance (Kbp)
from ZFP57 peak at ICRs
HP1cTKO
b
DNA Methylation changes at ICRs in HP1cTKO
c
Nnat
Genes
CpG Islands
CpG:_51
Input
ZFP57 ChIP
RRBS
WT
HP1cTKO
% meth.
KAP1
H3K9me3
H4K20me3
chr2:157,559,114-157,563,197
mm10
d
Protocadherin Cluster
α
β
γ
Genes
Gm6756
Pcdha1
Gm37013
Pcdhb16
Taf7
Pcdhga1
Pcdhgc5
CpG Islands
IAPEz-int
GSM2803559
WT Cortex H3K9me3
GSM2803563
WT Cortex H4K20me3
ESCs
KAP1
H3K9me3
H4K20me3
WT
HP1cTKO
chr18:36,764,128-37,874,122
mm10

Figure S4 (Previous Page)

H3K9me3 is lost at repeats and ICRs but is unchanged at regulatory regions of the protocadherin cluster in HP1cTKO ES cells. **a** Read coverage of KAP1, H3K9me3 and H4K20me3 centered at ZFP57 peaks found at mouse imprinting control regions (ICRs) averaged over biological replicates. (RPGC-normalized bigwigs were averaged, 50bp comatrices were used and profiles were plotted over ICRs with a window of 150bp). **b** DNA Methylation changes at ICRs in HP1cTKO are mixed but show a tendency towards 'hypermethylation' which would be consistent with increased 5hmC observed in figure S5a. **c** Genome browser view of changes occurring in HP1cTKO ESCs at imprinting control region found at Neuronatin (*Nnat*). In addition to shifts in DNA methylation seen in the RRBS track, KAP1 is lost in HP1cTKO ESCs surrounding the ICR (CpG island marked by ZFP57; ZFP57 ChIP data from [41]). **d** Genome browser view of changes to KAP1, H3K9me3 and H4K20me3 at the protocadherin locus in HP1cTKO ESCs with reference tracks for refseq genes, CpG islands, IAPEz-int, along with published ChIPseq of H3K9me3 and H4K20me3 from the cortex [40]. H3K9me3 and H4K20me3 mark regulatory domains over the protocadherin cluster in cortical neurons. At the same locus in embryonic stem cells (ESCs), H3K9me3 can be seen at IAPs and regulatory domains, and in HP1cTKO cells H3K9me3 is only lost at IAPs. H4K20me3 at the protocadherin cluster in wt ES cells is prominently observed at IAP loci (empty arrows) and at the proximal promoter (*), and is completely lost in HP1cTKO ESCs.

Figure S5

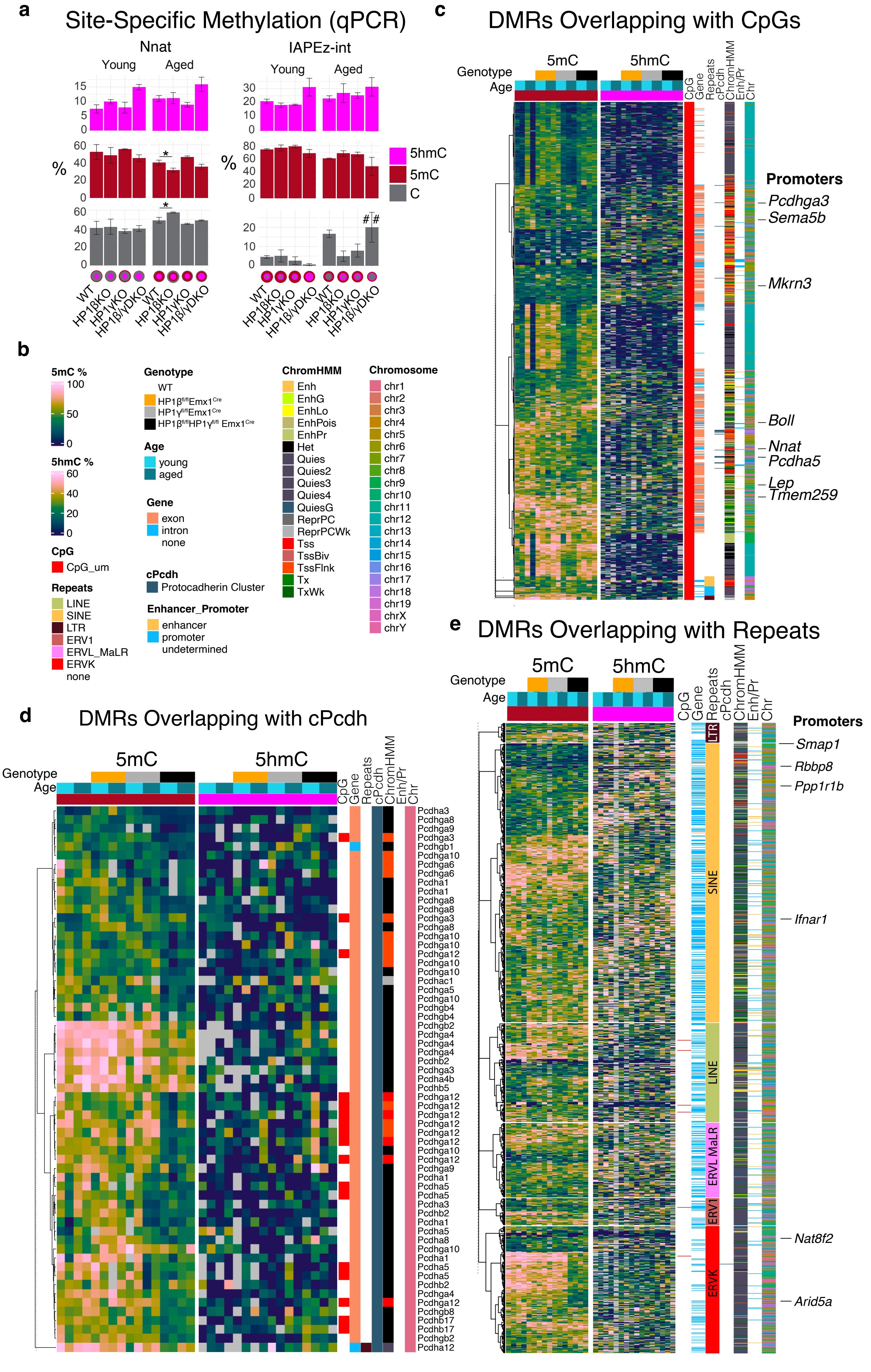

a
Site-Specific Methylation (qPCR)
Nnat
IAPEz-int
Young
Aged
%
5hmC
5mC
C
WT
HP1βKO
HP1γKO
HP1β/γDKO
b
5mC %
100
50
0
5hmC %
60
40
20
0
CpG
CpG_um
Repeats
LINE
SINE
LTR
ERV1
ERVL_MaLR
ERVK
none
Genotype
WT
HP1β fl/fl Emx1 Cre
HP1γ fl/fl Emx1 Cre
HP1β fl/fl HP1γ fl/fl Emx1 Cre
Age
young
aged
Gene
exon
intron
none
cPcdh
Protocadherin Cluster
Enhancer_Promoter
enhancer
promoter
undetermined
ChromHMM
Enh
EnhG
EnhLo
EnhPois
EnhPr
Het
Quies
Quies2
Quies3
Quies4
QuiesG
ReprPC
ReprPCWk
Tss
TssBiv
TssFlnk
Tx
TxWk
Chromosome
chr1
chr2
chr3
chr4
chr5
chr6
chr7
chr8
chr9
chr10
chr11
chr12
chr13
chr14
chr15
chr16
chr17
chr18
chr19
chrX
chrY
c
DMRs Overlapping with CpGs
Genotype
Age
5mC
5hmC
CpG
Gene
Repeats
cPcdh
ChromHMM
Enh/Pr
Chr
Promoters
Pcdhga3
Sema5b
Mkrn3
Boll
Nnat
Pcdha5
Lep
Tmem259
d
DMRs Overlapping with cPcdh
Genotype
Age
5mC
5hmC
CpG
Gene
Repeats
cPcdh
ChromHMM
Enh/Pr
Chr
Pcdha3
Pcdhga8
Pcdhga9
Pcdhga3
Pcdhgb1
Pcdhga10
Pcdhga6
Pcdhga6
Pcdha1
Pcdha1
Pcdhga8
Pcdhga8
Pcdhga3
Pcdhga8
Pcdhga10
Pcdhga10
Pcdhga12
Pcdhga10
Pcdhga10
Pcdhac1
Pcdhga5
Pcdhga10
Pcdhgb4
Pcdhgb4
Pcdhgb2
Pcdhga4
Pcdhga4
Pcdhga4
Pcdhb2
Pcdhga3
Pcdha4b
Pcdhb5
Pcdhga12
Pcdhga12
Pcdhga12
Pcdhga12
Pcdhga12
Pcdhga12
Pcdhga10
Pcdhga12
Pcdhga9
Pcdha1
Pcdha5
Pcdha5
Pcdha3
Pcdhb2
Pcdha1
Pcdha5
Pcdha8
Pcdhga10
Pcdha1
Pcdha5
Pcdha5
Pcdhb2
Pcdhga4
Pcdhga12
Pcdhgb8
Pcdhb17
Pcdhb17
Pcdhgb2
Pcdha12
e
DMRs Overlapping with Repeats
Genotype
Age
5mC
5hmC
CpG
Gene
Repeats
cPcdh
ChromHMM
Enh/Pr
Chr
LTR
SINE
LINE
ERVL MaLR
ERV1
ERVK
Promoters
Smap1
Rbbp8
Ppp1r1b
Ifnar1
Nat8f2
Arid5a

Figure S5 (Previous Page)

DNA methylation is affected in several regions in HP1 deficient hippocampi. **a** Profiles of qPCR-profiled cytosine hydroxymethylation (5hmC), cytosine methylation (5mC) and unmethylated cytosines (C) at selected CCGG sites in Neuronatin (*Nnat*) and IAPEz-int from hippocampal lysate DNA. Mean ±SEM; 3 biological replicates for each, two-way ANOVA, * = $p < 0.05$ within age across genotype, ## = $p < 0.01$ within genotype across age. **b** Legend for heatmaps in **c,d,e** for significant DMRs subset by row annotations corresponding to CpG islands, gene body, repeats, P0 Cortex ChromHMM state and chromosome. **c** DMRs (% difference > 25%, $q < 0.05$) overlapping annotated CpG islands. **d** DMRs (% difference > 25% $q < 0.05$) occurring in the protocadherin cluster. **e** DMRs (% difference > 30%, $q < 0.05$) that overlap an annotated repeat. DMR in this context is a single cytosine.

Figure S6

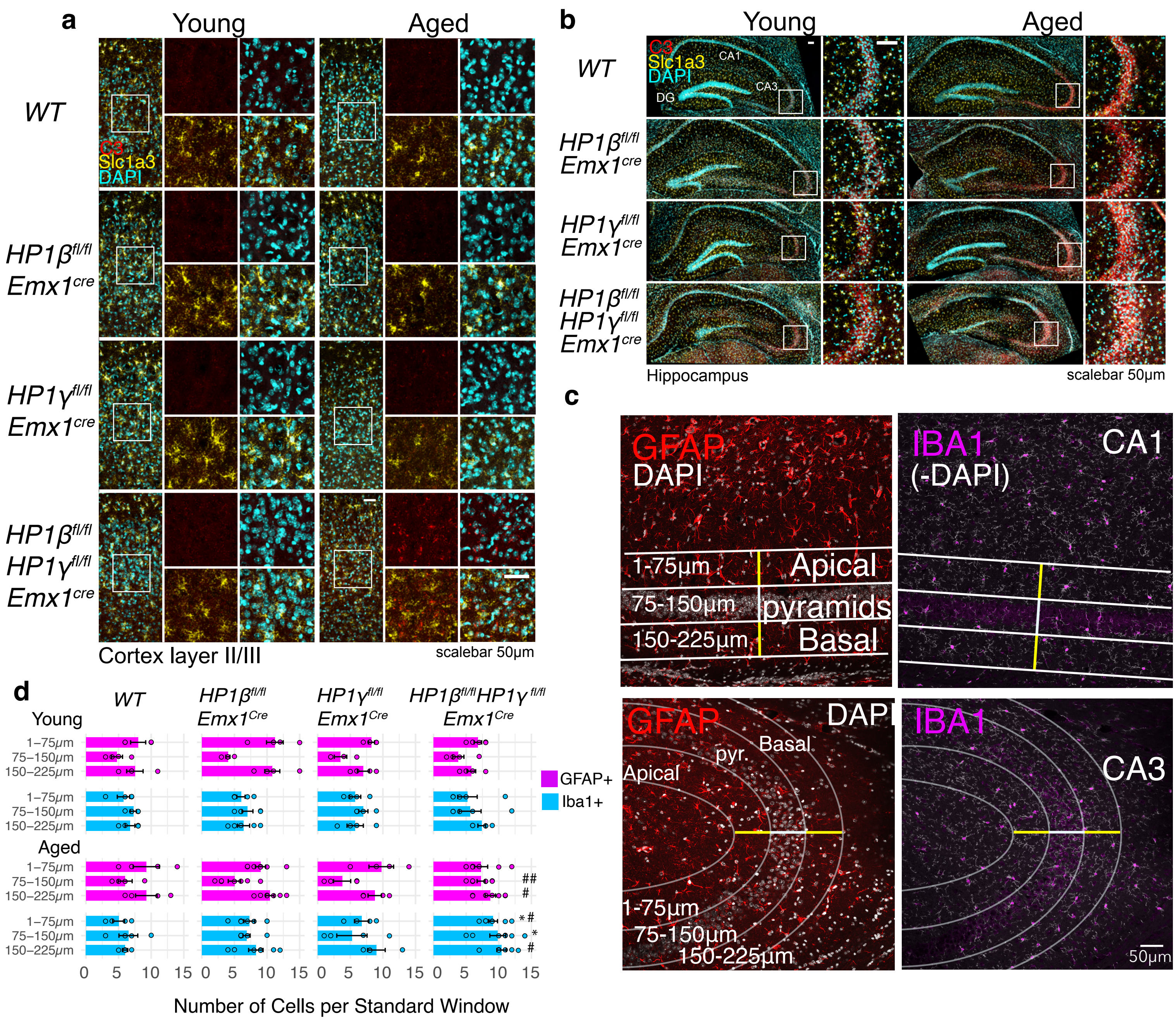

Figure S6. Initiation of Complement and invasion of microglia in HP1DKO cortices and hippocampi. Multiplex In situ hybridization using probes for C3 and Slc1a3 in the cortex (**a**) and hippocampus (b) of young and aged HP1 mutants. Distribution of astrocytes and microglia in or adjacent to pyramidal layers of the hippocampus. **c** Quantification scheme for CA1 and CA3 fields. Iba1+ microglia and GFAP+ cells with astrocyte morphology were quantified with 75 µm bins in standard windows across apical, pyramidal and basal fields. For CA1, straight lines separated by 75µm equivalent pixels was used, whereas for CA3, standard ellipses separated with 75µm equivalent pixels were used. Quantification summary of GFAP+ and Iba1+ can be seen by bin in **d** and across all fields in figure **5m**. Two way ANOVA followed by Tukey HSD posthoc correction on multiple comparisons. Plots denote asterisks (*) for significant changes compared to WT within age, and hashtags (#) for significant changes to young within genotype. All values and exact p values can be seen in the Source Data file.

Figure S7

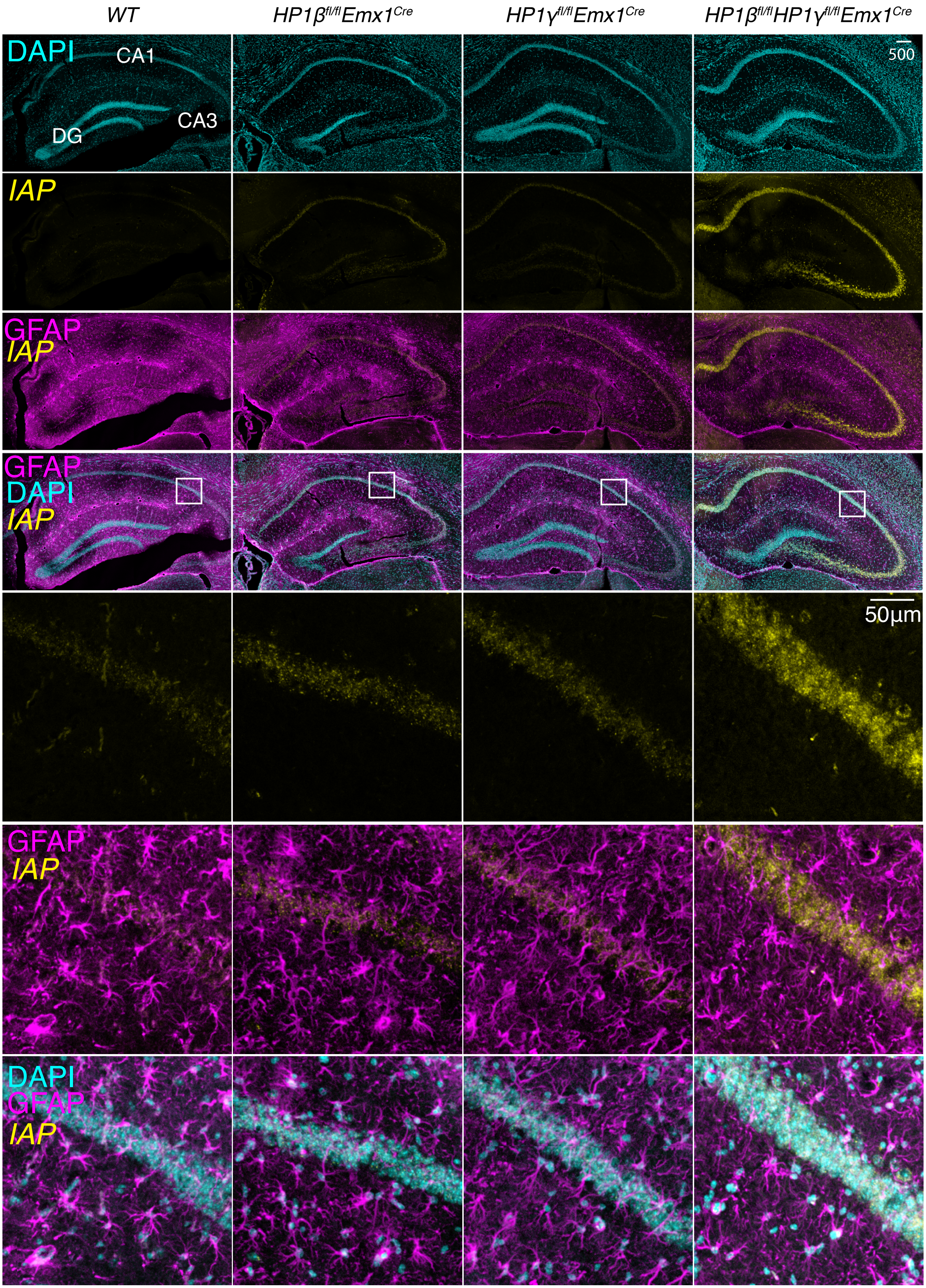

WT
HP1β fl/fl Emx1 Cre
HP1γ fl/fl Emx1 Cre
HP1β fl/fl HP1γ fl/fl Emx1 Cre
DAPI
CA1
CA3
DG
500
IAP
GFAP
IAP
GFAP
DAPI
IAP
50µm
GFAP
IAP
DAPI
GFAP
IAP

Figure S7 (Previous Page)

Emx1Cre-mediated deletion of HP1 in Neurons and Astrocytes results in *IAP* de-repression only in Neurons. Co-staining of *IAP* RNA transcripts (RNAscope, yellow), GFAP (Antibody, Magenta), and DAPI (Cyan) in aged hippocampi from WT and HP1 single and double mutants.

Figure S8

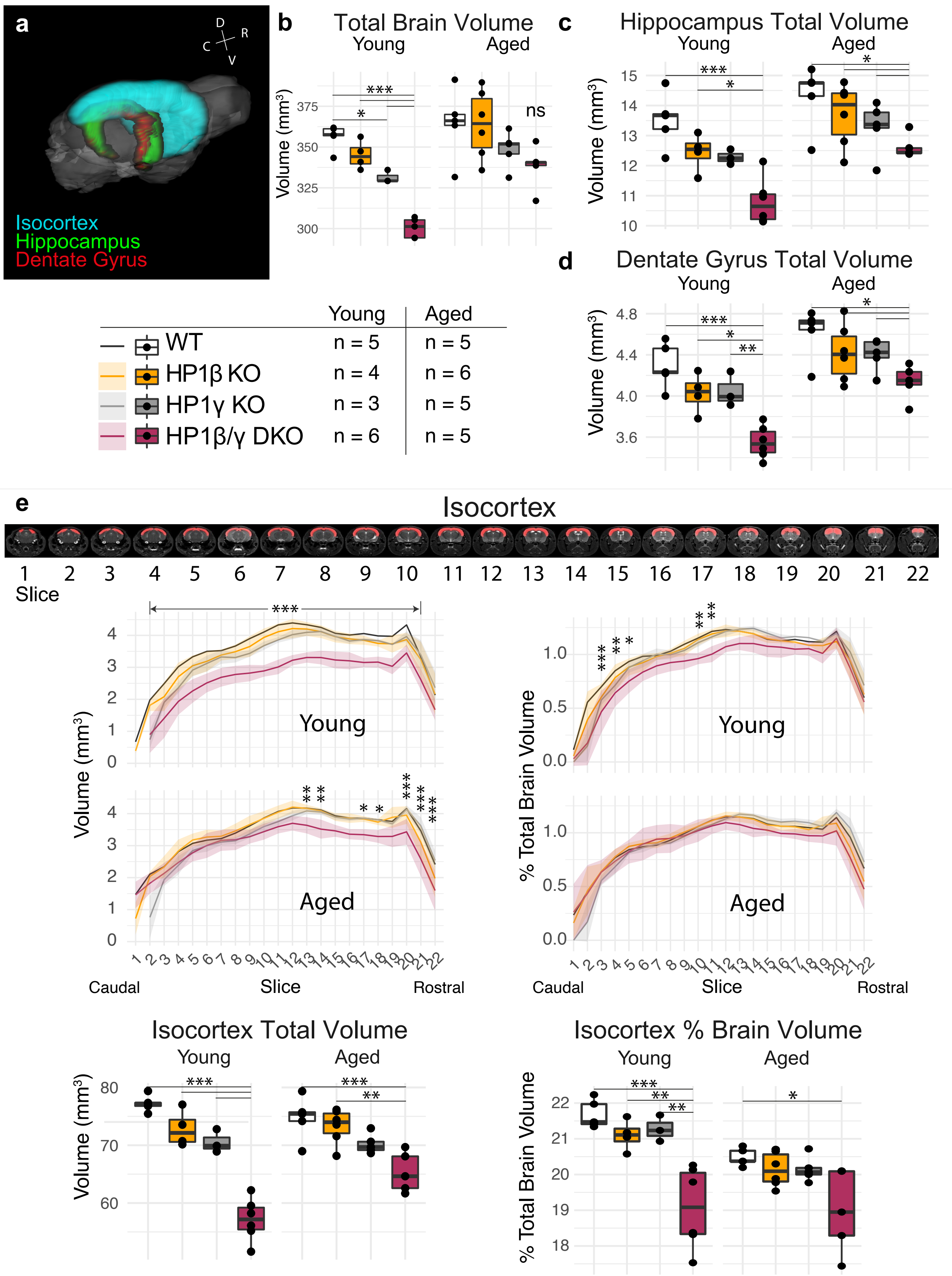

a
D
R
C
V
Isocortex
Hippocampus
Dentate Gyrus
b
Total Brain Volume
Young
Aged
Volume (mm³)
375
350
325
300
***
*
ns
c
Hippocampus Total Volume
Young
Aged
Volume (mm³)
15
14
13
12
11
10
***
*
*
d
Dentate Gyrus Total Volume
Young
Aged
Volume (mm³)
4.8
4.4
4.0
3.6
***
*
**
*
Young
Aged
WT n = 5 n = 5
HP1β KO n = 4 n = 6
HP1γ KO n = 3 n = 5
HP1β/γ DKO n = 6 n = 5
e
Isocortex
1 2 3 4 5 6 7 8 9 10 11 12 13 14 15 16 17 18 19 20 21 22
Slice
***
Young
Aged
Volume (mm³)
% Total Brain Volume
Caudal
Slice
Rostral
Isocortex Total Volume
Young
Aged
Volume (mm³)
80
70
60
***
**
Isocortex % Brain Volume
Young
Aged
% Total Brain Volume
22
21
20
19
18
***
**
**
*

Figure S8 (Previous Page)

Structural Magnetic Resonance Imaging of HP1 mutants. **a** Registration of Isocortex, Hippocampus and Dentate Gyrus. Absolute volumes of total brain **b**, Hippocampus **c** and dentate gyrus **d** in young and aged HP1 mutants. **e** Slice-wise depiction of isocortical volume, both absolute (left) and as a percentage of total brain volume (right). All animals were included in the analysis. Statistics: one-way ANOVA within age with Bonferroni multiple comparison corrections. *** = $p < 0.001$, ** = $p < 0.01$, * = $p < 0.05$. All values and exact p values can be seen in the Source Data file.

Figure S9

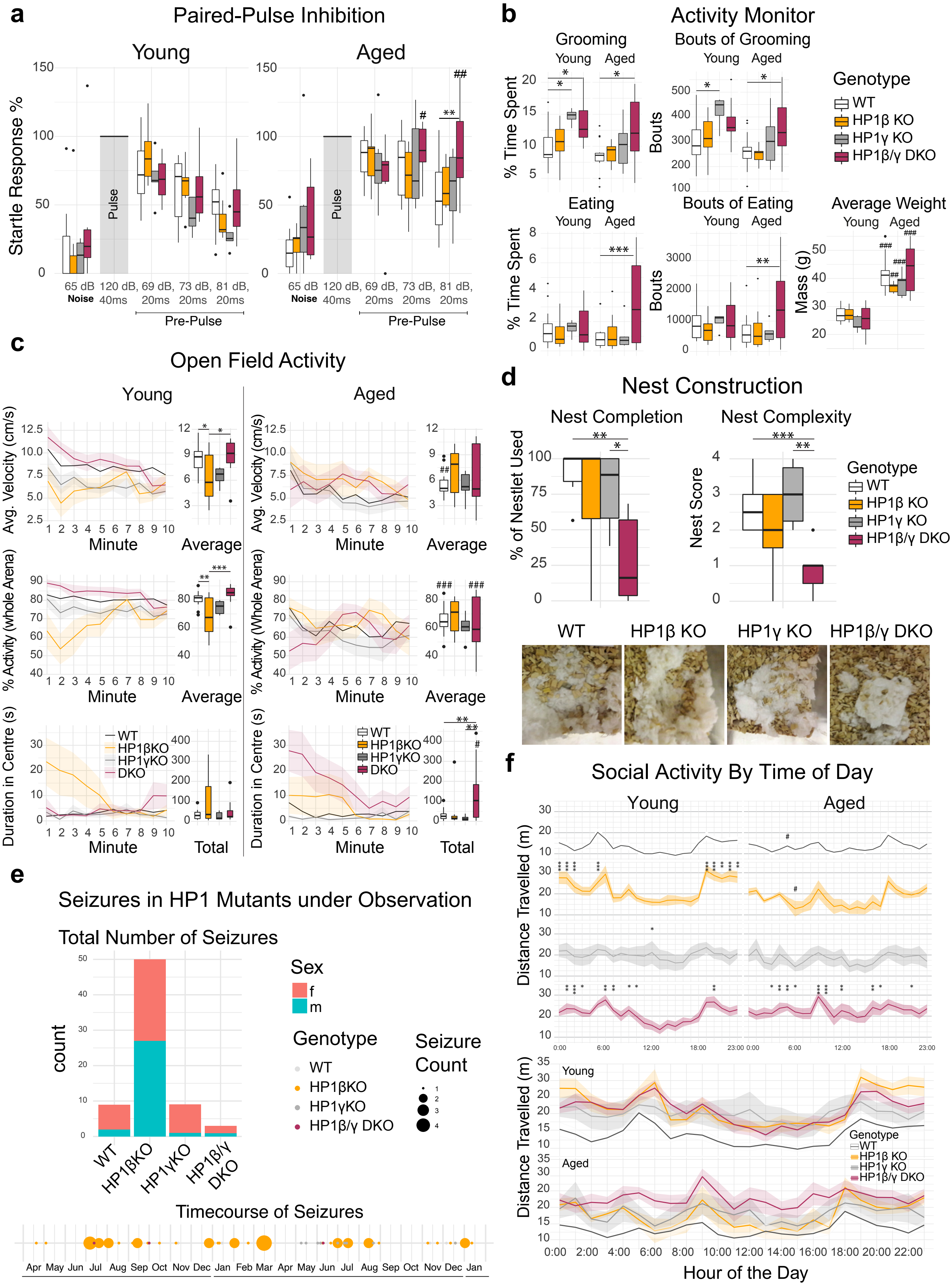
a
Paired-Pulse Inhibition
Young
Aged
Startle Response %
Pulse
65 dB Noise
120 dB, 40ms
69 dB, 20ms
73 dB, 20ms
81 dB, 20ms
Pre-Pulse
b
Activity Monitor
Grooming
Bouts of Grooming
% Time Spent
Bouts
Eating
Bouts of Eating
Average Weight
Mass (g)
Genotype
WT
HP1β KO
HP1γ KO
HP1β/γ DKO
c
Open Field Activity
Avg. Velocity (cm/s)
% Activity (whole Arena)
Duration in Centre (s)
Minute
Average
Total
WT
HP1βKO
HP1γKO
DKO
d
Nest Construction
Nest Completion
Nest Complexity
% of Nestlet Used
Nest Score
WT
HP1β KO
HP1γ KO
HP1β/γ DKO
e
Seizures in HP1 Mutants under Observation
Total Number of Seizures
count
Sex
f
m
Genotype
WT
HP1βKO
HP1γKO
HP1β/γ DKO
Seizure Count
Timecourse of Seizures
Apr May Jun Jul Aug Sep Oct Nov Dec Jan Feb Mar Apr May Jun Jul Aug Sep Oct Nov Dec Jan
f
Social Activity By Time of Day
Young
Aged
Distance Travelled (m)
Genotype
WT
HP1β KO
HP1γ KO
HP1β/γ DKO
0:00 2:00 4:00 6:00 8:00 10:00 12:00 14:00 16:00 18:00 20:00 22:00
Hour of the Day

Figure S9 (Previous Page)

Age-related changes in behavior in HP1β/γDKO animals. **a** Paired-Pulse Inhibition recordings across HP1 mutants. All paired startle responses are percent normalized to the amplitude of the startle pulse (120dB) alone. A strong age-dependent change in paired pulse inhibition was seen in HP1β/γDKO animals, where pre-pulses of larger amplitude failed to further attenuate a startle response. **b** Altered Grooming, Eating and Circadian Activity in HP1mutants. HP1DKO animals showed an increased total time and frequency of grooming, and aged HP1DKO also displayed higher bouts of eating, though this did not translate into an increase in weight. Anxiety and exploration measured in an open field test revealed age dependent delays in HP1DKO animals (**c**). Young HP1βKO animals displayed non-convulsive freezing responses upon placement in the chamber which bias their measurements. **d** Nest construction profiled in aged animals was scored based on nest completion (% Nestlet used) and nest complexity (standard rubric, see methods) show marked deficits in HP1DKO animals. **e** Seizures observed in HP1 animals occurred predominantly in HP1β KO animals and were typically stimulation dependent, where observed seizures were non-randomly distributed, often occurring because of standard cage changes. Social activity **f** of mice observed over a 14 day period binned by hour reveals general hyperactivity in HP1βKO and HP1β/γDKO animals as well as an age-dependent change in peak nocturnal activity. Box and whiskers plot median, with lower hinge at the 25th percentile, upper hinge at the 75th percentile, and whiskers extending to the outer datapoint when within 1.5* the interquartile range. Statistics are two-way ANOVA with Bonferroni correction on multiple comparisons; Asterisks (*) denote tests to wildtype within age, hashtags (#) denote tests within genotype between age. All values and exact p values can be seen in the Source Data file.

Figure S10

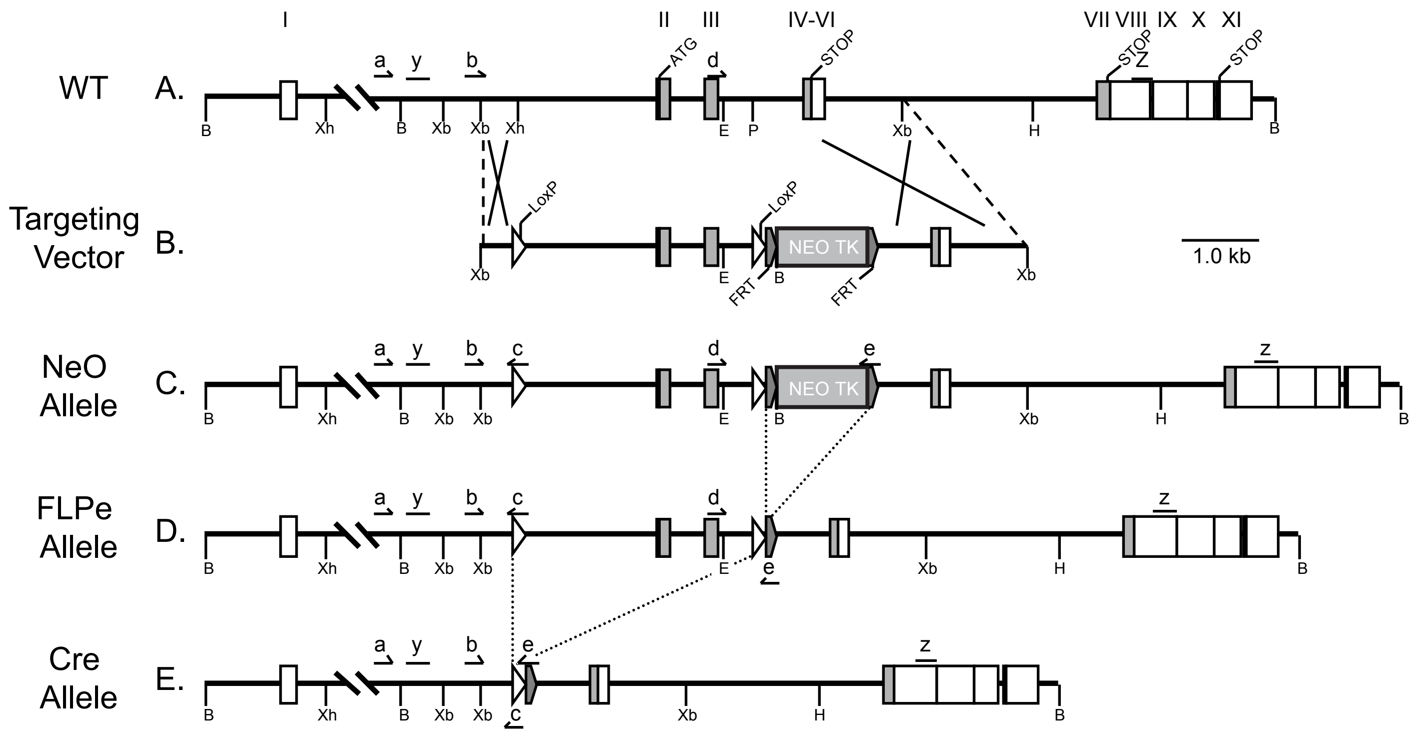

Figure S10 Targeting of CBX1 (HP1β) Genomic Locus for generation of HP1FEC mouse

Abbreviations to indicate restriction sites and primers:

B: BamHI	P: PstI	a: primer M31XbaS	d: primer M31PstS
E: EcoRI	Xb: XbaI	b: primer M31loxPS	e: primer FRTa
H: HindIII	Xh: XhoI	c: primer M31loxA2	

Figure S11

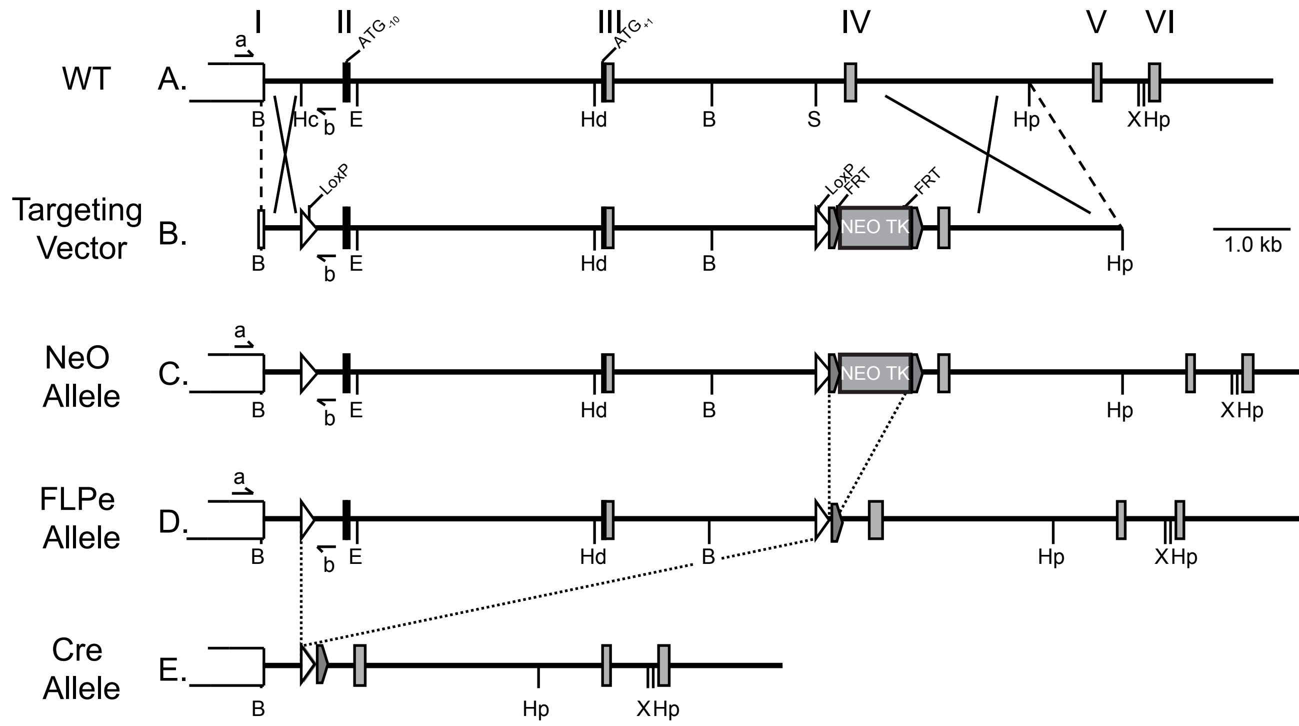

Figure S11 Targeting of CBX3 (HP1γ) Genomic Locus for generation of HP1FEC mouse

Abbreviations to indicate restriction sites:

B: BamHI Hp: HpaI Hc: HincII X: XhoI
E: EcoRI S: StuI Hd: HindIII

M. Nakayama KDRI

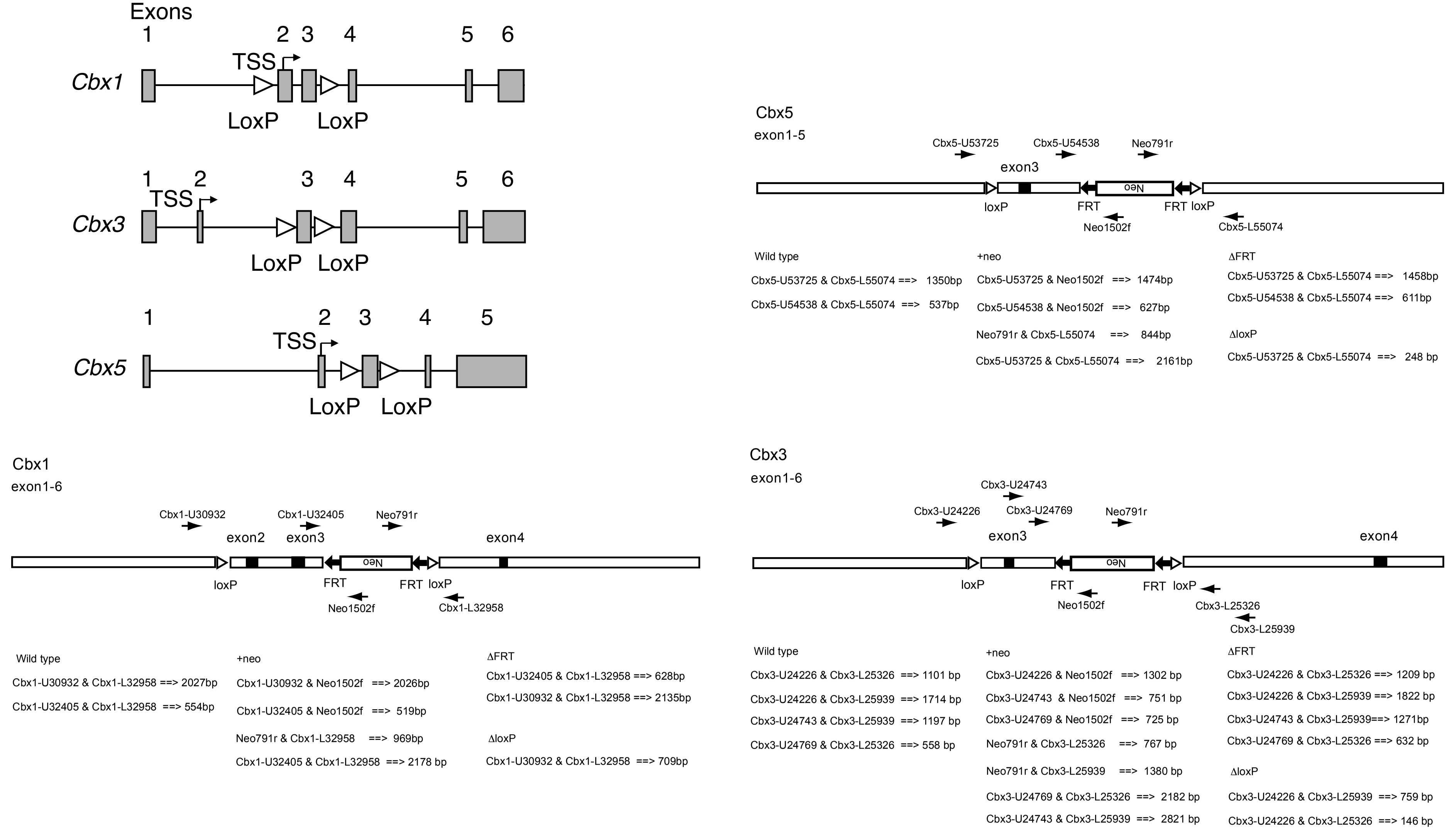

Figure S12 Targeting of Cbx1, Cbx3, & Cbx5 alleles in HP1cTKO ESCs

Full blots from Co-immunoprecipitation of SUV420H2myc with HP1-GFP proteins from Figure 2d.

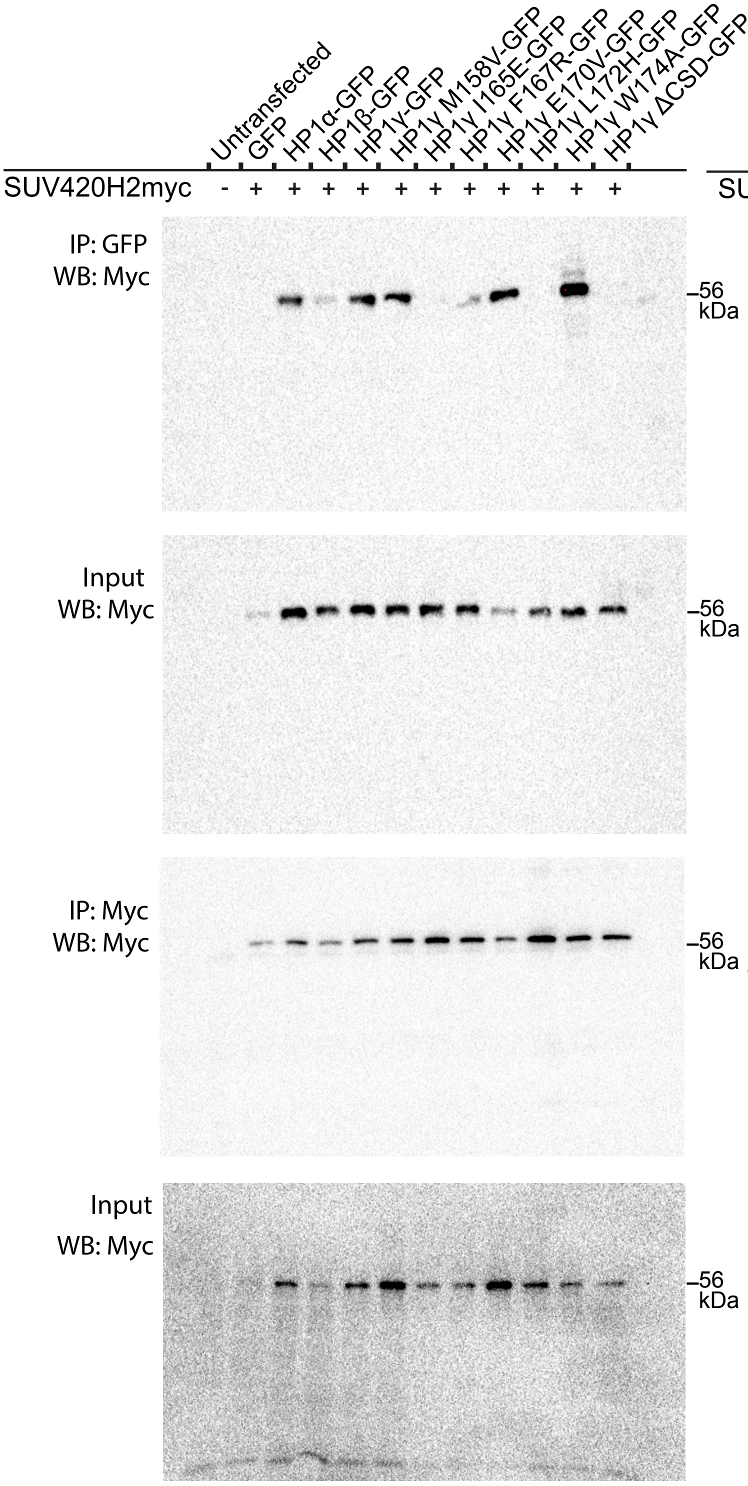

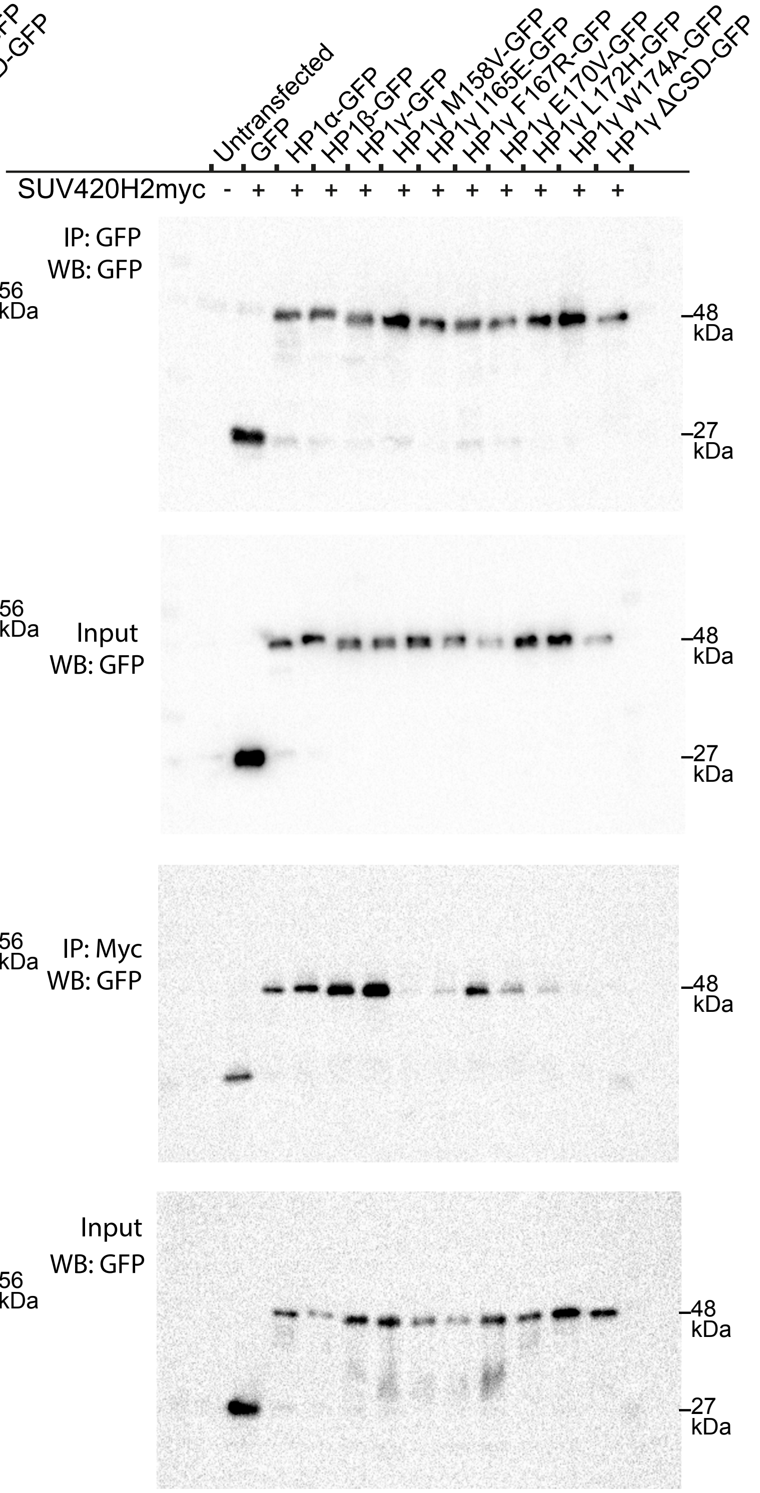

Full Western Blots from Figure 6

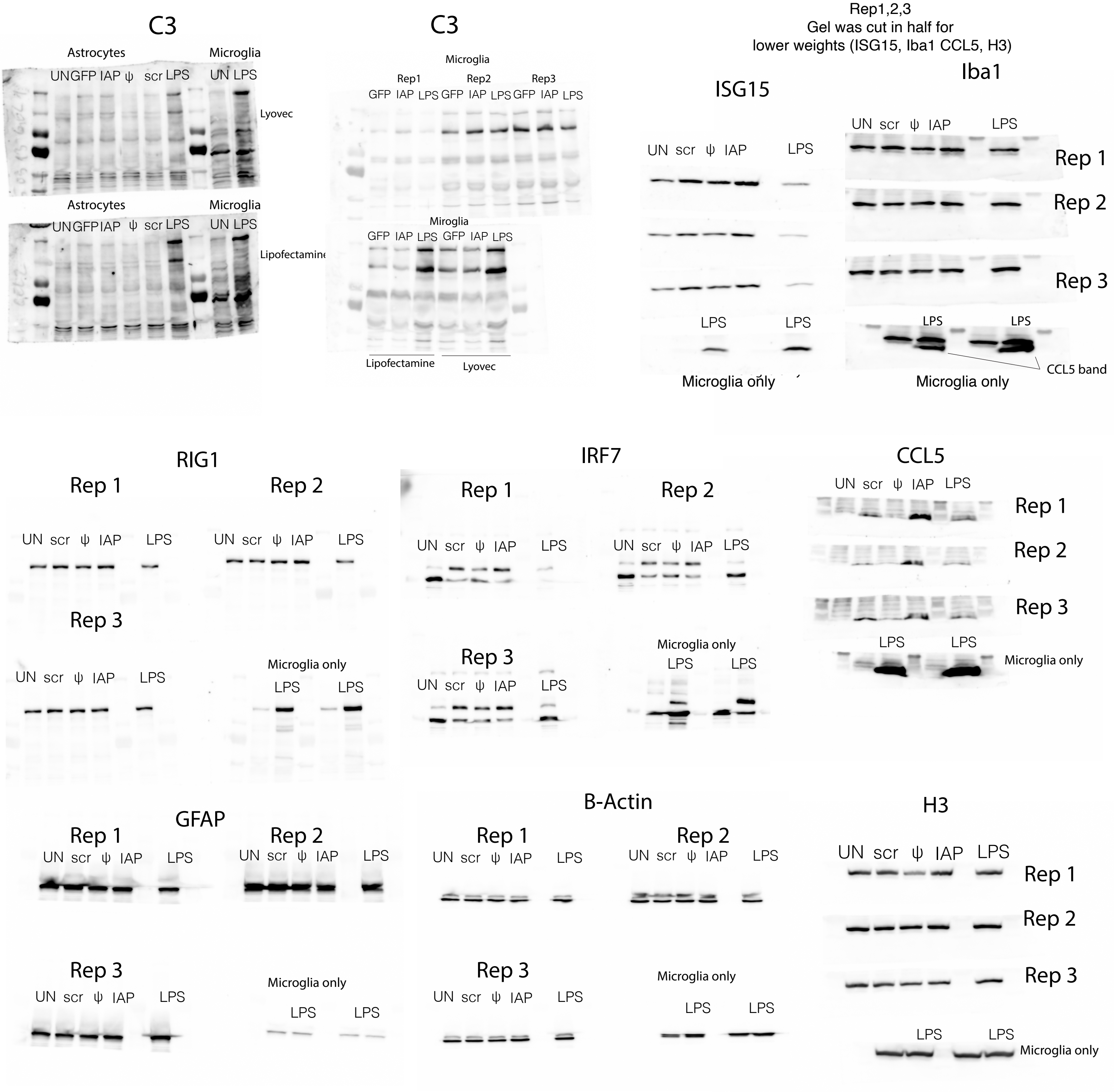

Supplement: Supplementary file 1 — Supplementary Information [file 41467_2025_61319_MOESM1_ESM.pdf]
